# Geographic and Program Size Disparities in Medicare Funding for Graduate Medical Education

**DOI:** 10.7759/cureus.111083

**Published:** 2026-06-18

**Authors:** Aryan Gupta, Prabhat K Gottipati, Priya Joshi, Medha Tekriwal, Subodha Kumar, Yoshiya Toyoda, Suyog A Mokashi

**Affiliations:** 1 Cardiovascular Surgery, Lewis Katz School of Medicine, Philadelphia, USA; 2 Statistics, Carnegie Mellon University, Pittsburgh, USA; 3 Statistics, Operations, and Data Science, Fox School of Business at Temple University, Philadelphia, USA

**Keywords:** geographic disparities, graduate medical education, healthcare workforce, medicare direct graduate medical education, residency training

## Abstract

Background

Medicare direct graduate medical education (DGME) payments are essential to sustaining residency training and shaping the physician workforce. While geographic variation in total DGME spending is well described, less is known about inequities in per-resident funding across states and program sizes.

Objective

The objective of this article was to assess variation in Medicare DGME payments per resident across US states and residency program sizes and identify patterns relevant to equitable graduate medical education (GME) funding.

Methods

In 2025, we conducted a retrospective cross-sectional analysis of publicly available Medicare DGME payment data from 2014-2024. All US hospitals reporting DGME payments were eligible (N = 447), with 439 hospitals included (98.2%). Hospitals were stratified by state and residency program size: small (0-20 residents), mid-sized (21-200), and large (>200). The primary outcome was DGME payment per resident. Comparisons were performed across states and program sizes. Sensitivity analyses evaluated resident count and hospital bed capacity.

Results

Per-resident DGME payments varied significantly by state and program size (p<0.001). The national median payment was approximately $22,000 per resident, with the highest payments in Mississippi and the lowest in California. Small programs received higher mean per-resident payments than mid-sized programs (p<0.01). Hospitals in the highest and lowest payment deciles were concentrated within specific states. Resident count was a stronger predictor of per-resident payments than hospital bed capacity (p<0.001).

Conclusions

Substantial geographic and program-size-related inequities in per-resident DGME funding persist nationwide. This may disadvantage mid-sized and underserved programs and should be considered in promoting equitable training and workforce distribution.

## Introduction

Graduate medical education (GME) is central to the preparation of the physician workforce that underpins the US healthcare system. Sustained investment in GME is essential to maintaining access to high-quality care as healthcare needs evolve in response to population aging and persistent regional disparities in physician availability. The structure and distribution of GME funding influence where physicians are trained and ultimately practice, making Medicare's role as the primary federal funder of residency training highly consequential for workforce distribution and the long-term sustainability of residency programs nationwide.

Medicare supports GME in part through direct graduate medical education (DGME) payments, which are intended to offset the direct costs of training resident physicians [1]. These costs include resident salaries and benefits, faculty teaching time, and administrative expenses. DGME payments are calculated on a per-resident basis using hospital-specific cost amounts, the number of full-time equivalent residents, and the proportion of inpatient care attributable to Medicare beneficiaries. The per-resident cost amounts used in these calculations are largely derived from historical data. Although periodic adjustments have been made, these amounts have not fully kept pace with contemporary training costs or regional variation in the cost of living.

Prior research has demonstrated substantial variation in total DGME payments across hospitals and states, even among institutions with similar training responsibilities [2]. These differences are largely attributed to historical funding baselines rather than contemporary program characteristics, resulting in persistent heterogeneity in federal support for residency training nationwide. Such variability has raised longstanding concerns regarding the equity of Medicare's DGME allocation framework, with early policy critiques noting that legacy-based payment structures may advantage certain regions independent of training volume or program scale [3]. While existing studies have characterized aggregate DGME spending patterns, far less attention has been given to variation in Medicare support on a per-resident basis. In particular, it remains unclear whether hospitals with comparable resident counts receive different levels of per-resident DGME funding based on geographic location. Moreover, prior analyses have not systematically examined how DGME payments vary across residency program size strata, such as small, mid-sized, and large programs, leaving an important dimension of funding equity insufficiently explored.

Disparities in per-resident DGME payments could have meaningful implications for the viability and growth of residency programs, particularly smaller and mid-sized programs that often operate with limited financial margins. Many such programs serve regions with limited physician supply, where training capacity plays an important role in workforce availability. Unequal funding may constrain the ability of these programs to expand resident positions, invest in educational infrastructure, or recruit faculty, potentially contributing to persistent geographic differences in physician training capacity.

To address these gaps in the literature, this study examined national Medicare DGME payment data from 2014 to 2024 to evaluate variation in per-resident funding across states and residency program sizes. The primary objective was to assess variation in per-resident DGME payments across US states. Secondary objectives were to evaluate differences in per-resident DGME payments across residency program size categories and identify states with the highest and lowest levels of per-resident DGME funding. By characterizing these patterns, this study aims to inform ongoing policy discussions regarding equitable GME funding and the sustainability of diverse residency training programs nationwide.

This work was presented as a poster at the 2026 ACGME Annual Educational Conference. 

## Materials and methods

Study design and data sources

We conducted a retrospective, cross-sectional analysis of national Medicare DGME payment data spanning fiscal years 2014 through 2024. DGME payment information was obtained from publicly available Centers for Medicare & Medicaid Services (CMS) hospital cost report files. Hospitals reporting DGME payments during the study period were identified, and hospital-level variables including total DGME payments, DGME part A/C payments, DGME part B payments, the number of full-time equivalent interns and residents, teaching status, and hospital bed capacity were extracted for analysis.

Hospitals reporting DGME payments during the study period were eligible for inclusion. Facilities with missing values for DGME payment components, resident counts, or hospital bed capacity were excluded from analysis. Missing data were handled using complete-case analysis, with only hospitals containing complete data for all study variables retained in the final analytic cohort. Hospitals reporting zero residents were retained only if DGME payments were reported, consistent with CMS accounting practices. Hospital-level characteristics were obtained from the same CMS cost report data to ensure consistency across sources and to support sensitivity analyses. This study was conducted in 2025.

Exposure variables and outcomes

The primary exposures of interest were geographic location and residency program size. Hospitals were grouped by US state to assess geographic variation in DGME payments. Residency program size was categorized a priori into three groups based on total resident count: small programs (0-20 residents), mid-sized programs (21-200 residents), and large programs (>200 residents). The primary outcome was DGME payment per resident, calculated by dividing total DGME payments by the number of reported interns and residents. Secondary outcomes included per-resident DGME part A/C and part B payments.

Statistical analysis

Descriptive statistics were used to summarize DGME payments per resident across states and residency program size categories. As DGME payment and resident count variables demonstrated substantial right skew, winsorization was applied at the 1st and 99th percentiles to reduce the influence of extreme values and improve model stability. A Box-Cox transformation was then used to normalize skewed variables for statistical testing, while untransformed values were retained for reporting and interpretation.

Comparisons of per-resident DGME payments across states and program size categories were performed using analysis of variance (ANOVA). Targeted state-level comparisons were conducted for selected pairs of states to illustrate differences in payment patterns across program sizes. Sensitivity analyses assessed the relative contribution of resident count and hospital bed capacity to variation in DGME payments per resident using linear regression models. All analyses were conducted using R statistical software (R Foundation, Vienna, Austria), with a two-sided p < 0.05 considered statistically significant. This study used publicly available, deidentified administrative data and was determined to be exempt from institutional review board review.

## Results

Between 2014 and 2024, 447 hospitals reported DGME payments to CMS. After excluding hospitals with missing DGME payments, resident counts, or bed capacity, the final analytic sample consisted of 439 hospitals. Across all included hospitals, the national median total DGME payment per resident was approximately $22,000, with substantial dispersion observed across states and program sizes.

**Figure 1:**
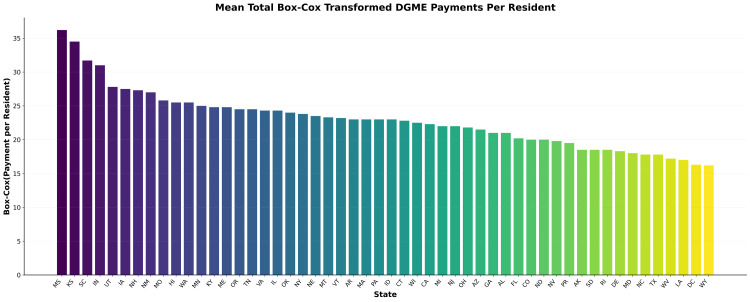
Geographic variation in per-resident DGME payments by state Mean per-resident Medicare direct graduate medical education (DGME) payment by US state, calculated as total DGME payments divided by the number of reported interns and residents, and Box-Cox transformed to reduce skewness. Substantial geographic variation is observed, with several states exhibiting consistently higher or lower per-resident payments.

Per-resident DGME payments varied widely across US states. State-level median payments ranged from approximately $30,000 per resident in Mississippi to approximately $16,600 in the District of Columbia and $15,000 in California. Similar patterns of geographic variation were observed across DGME payment components, including total DGME, Part A/C, and Part B payments, indicating that state-level differences persisted across both hospital-based and physician-related funding streams. Mississippi and Kansas consistently ranked among the highest states in per-resident DGME payments, whereas the District of Columbia, Louisiana, and West Virginia were among the lowest funded states on a per-resident basis. Median per-resident DGME payments are reported for descriptive state-level comparisons, while mean values (Box-Cox transformed) are presented in figures and regression analyses to facilitate modeling and visualization (Figure 1).

**Figure 2:**
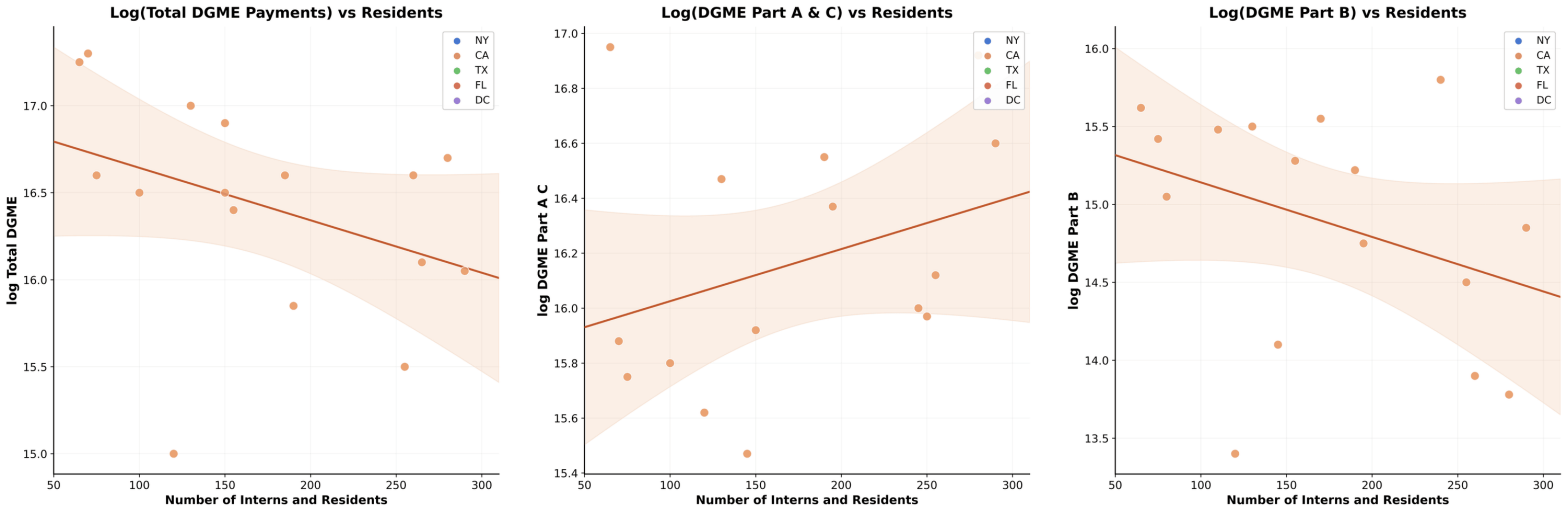
Association between residency program size and per-resident DGME payments Scatterplots showing the relationship between the number of interns and residents and per-resident direct graduate medical education (DGME) payments for total DGME, part A/C, and part B components. Fitted regression lines with 95% confidence intervals demonstrate non-proportional scaling of per-resident payments with program size.

Per-resident DGME payments also differed by residency program size. Small programs (0-20 residents) received significantly higher mean per-resident DGME payments than mid-sized programs (21-200 residents) (p<0.01). Large programs (>200 residents) demonstrated modestly higher per-resident payments relative to mid-sized programs, though greater variability was observed within this group (Figure 2). This pattern was consistent across total DGME, part A/C, and part B payment components, indicating that differences by program size were not confined to a single payment category.

Targeted state-level comparisons demonstrated that the relationship between geography and per-resident DGME payments varied by program size. Relative differences in per-resident funding between states were not uniform across small, mid-sized, and large programs, indicating that state-level funding advantages did not persist consistently across program size strata. These findings suggest that geographic variation in DGME payments interacts with residency program size rather than operating independently.

When hospitals were stratified into the highest and lowest deciles of per-resident DGME payments, these extremes were not evenly distributed across states. Hospitals in both the highest and lowest per-resident DGME payment deciles were disproportionately concentrated within specific states, rather than evenly distributed across the country. This concentration pattern was observed across total DGME, part A/C, and part B payments, indicating that extremes of per-resident funding were consistent across DGME components.

In sensitivity analyses examining predictors of per-resident DGME payments, resident count demonstrated a stronger association with per-resident payments than hospital bed capacity (p<0.001). This relationship was consistent across DGME payment components, indicating that variation in per-resident funding was more closely associated with the size of the residency workforce than with overall hospital capacity.

## Discussion

This analysis demonstrates that Medicare direct graduate medical education (DGME) payments vary substantially on a per-resident basis across US states and residency program sizes. These differences persist even among hospitals with similar resident counts, highlighting a misalignment between DGME funding levels and training volume and extending prior descriptions of geographic variation in GME financing. This variation is observed across total DGME, part A/C, and part B components, suggesting that the disparities reflect structural features of the DGME allocation framework rather than institution-specific anomalies. 

This per-resident framework indicates that DGME allocations do not scale proportionally with training load, extending prior work documenting variation in GME funding across institutions and regions [4]. State-level patterns in per-resident DGME payments persisted across the study period and were consistent across DGME components, with certain states exhibiting systematically higher or lower funding levels. These findings align with prior descriptions of geographic variation in Medicare GME financing, which emphasize the enduring influence of historically derived payment structures on contemporary funding patterns [5]. Notably, observed differences in per-resident DGME payments were not explained by resident count alone, indicating that geographic location remains an independent correlate of DGME funding. Together, these findings indicate that legacy-based per-resident payment amounts derived from historical cost reports continue to shape present-day funding distributions across states, reinforcing prior observations that geography influences GME training resources independent of program characteristics or performance measures [6]. In addition to geographic variation, residency program size was associated with differences in per-resident DGME payments. Small programs received higher per-resident payments than mid-sized programs, while the largest programs demonstrated modest increases, consistent with nonlinear scaling of DGME funding relative to program size. One potential explanation is that small programs distribute fixed educational and administrative costs across fewer trainees, whereas very large programs may benefit from institutional leverage and economies of scale [7]. In contrast, mid-sized programs may lack these structural advantages, contributing to comparatively lower per-resident DGME support within the current funding framework.

This analysis suggests that mid-sized residency programs receive lower per-resident DGME support relative to both small and large programs, a pattern that warrants consideration given the role these institutions play in the US training landscape. Mid-sized programs frequently function as regional referral and training hubs and are often located in mixed urban-rural or underserved areas. Prior work has shown that such programs contribute substantially to training physicians who practice outside major academic centers [8]. Although causal relationships cannot be inferred from the present analysis, comparatively lower per-resident DGME support may constrain flexibility for program growth or investment in educational infrastructure. Importantly, geography and program size jointly influenced DGME payment patterns. State-level funding advantages did not persist uniformly across program size strata, as relative differences in per-resident funding varied depending on whether programs were small, mid-sized, or large. This interaction suggests that funding disparities are not explained by geography or program size alone. Prior federal efforts to redistribute residency positions did not explicitly account for these combined effects and resulted in limited allocation of new positions to rural and mid-sized teaching hospitals [8].

This analysis also identified marked polarization in per-resident DGME payments, with hospitals in the highest and lowest payment deciles disproportionately represented within certain states. These patterns indicate that extremes of DGME funding are not evenly distributed nationally and may persist across hospitals within particular geographic areas over time. Given that physicians often practice near the locations where they complete residency training, sustained disparities in GME funding may have downstream implications for regional workforce distribution, particularly in underserved or rural areas [9].

These findings are particularly relevant in the context of ongoing national discussions surrounding GME reform and residency expansion. Prior efforts to expand residency positions without addressing underlying funding inequities have demonstrated limited effectiveness in redistributing training capacity to under-resourced regions [8]. Taken together, the observed polarization in per-resident DGME payments suggests that baseline funding distributions warrant consideration in future GME expansion efforts to avoid reinforcing existing disparities. 

These findings should be interpreted in light of several important limitations. As a retrospective observational analysis, this study cannot establish causal relationships between DGME funding levels and program-level or workforce outcomes. CMS hospital cost report data may be subject to reporting variability, and this analysis could not directly account for program quality, educational outcomes, or regional differences in cost of living. In addition, hospital-level characteristics such as ownership structure, urban-rural location, institutional resources, and local labor market conditions may influence DGME funding patterns and were not captured in the available dataset. Future research should build on these findings by incorporating cost-of-living adjustments and examining links between DGME funding patterns and downstream workforce outcomes, such as resident retention, specialty selection, and geographic practice patterns. Simulation of alternative DGME allocation models may also help inform discussions regarding how different funding frameworks could support a more balanced distribution of residency training capacity.

## Conclusions

In this national analysis of Medicare direct graduate medical education funding, substantial variation in per-resident DGME payments was observed across US states and residency program sizes. These differences persisted across DGME payment components and were not fully explained by resident count alone, indicating that structural features of the DGME allocation framework continue to shape funding distributions. Per-resident normalization revealed geographic and program-size-related disparities that are not apparent in aggregate spending analyses.
